# Myokine Levels in Relation to Bone Markers and Adipokines in Children with Prader–Willi Syndrome During Growth Hormone Therapy and Dietary Intervention

**DOI:** 10.3390/ijms262210822

**Published:** 2025-11-07

**Authors:** Joanna Gajewska, Magdalena Chełchowska, Katarzyna Szamotulska, Małgorzata Strucińska, Witold Klemarczyk, Jadwiga Ambroszkiewicz

**Affiliations:** 1 Department of Screening Tests and Metabolic Diagnostics, Institute of Mother and Child, 01-211 Warsaw, Poland; magdalena.chelchowska@imid.med.pl (M.C.); jadwiga.ambroszkiewicz@imid.med.pl (J.A.); 2 Department of Epidemiology and Biostatistics, Institute of Mother and Child, 01-211 Warsaw, Poland; katarzyna.szamotulska@imid.med.pl; 3 Department of Nutrition, Institute of Mother and Child, 01-211 Warsaw, Poland; malgorzata.strucinska@imid.med.pl (M.S.); witold.klemarczyk@imid.med.pl (W.K.)

**Keywords:** Prader–Willi syndrome, irisin, myostatin, osteocalcin, insulin-like growth factor-I, IGF-binding protein-2, body composition

## Abstract

Data on the interplay between muscle, bone, and adipose tissue metabolism in normal-weight children with Prader–Willi syndrome (PWS) undergoing growth hormone (GH) therapy and dietary interventions are limited. This study aimed to assess the myokine profile and explore the associations between myokines, bone markers, adipokines, and body composition in these patients. The study included 26 children with PWS and 26 age-matched healthy controls. Serum levels of irisin, myostatin (MSTN), fibroblast growth factor-2, insulin-like growth factor-I (IGF-I), IGF-binding protein-2, bone alkaline phosphatase (BALP), osteocalcin (OC), carboxylated OC (Gla-OC), periostin, soluble receptor activator of nuclear factor kappa-B ligand, tartrate-resistant acid phosphatase 5b, leptin/soluble leptin receptor, adiponectin, and proinsulin were measured using immunoenzymatic assays. Children with PWS had significantly lower lean mass (*p* = 0.047) and a higher fat mass/lean mass ratio (*p* < 0.001) than controls. Irisin levels were lower in the PWS group (*p* = 0.031), while MSTN levels were similar between the groups. In patients, irisin positively correlated with BALP (*p* = 0.025) and negatively correlated with Gla-OC (*p* = 0.041) and periostin (*p* = 0.005). MSTN was positively associated with proinsulin (*p* = 0.001) and negatively associated with lean mass (*p* = 0.015). OC concentration was lower in the PWS group and correlated positively with lean mass (*p* = 0.052). Children with PWS exhibit altered myokine, osteokine, and adipokine profiles, as well as differences in body composition. Reduced irisin and osteocalcin levels, along with the negative association between MSTN and lean mass, may impair muscle development and bone metabolism. These imbalances could also contribute to future metabolic disorders in patients with PWS.

## 1. Introduction

Prader–Willi syndrome (PWS) is a complex genetic disorder characterized by distinct phenotypic and metabolic features, including endocrine disorders, growth hormone (GH) deficiency, short stature, hyperphagia, morbid obesity, bone impairment, hypotonia, and muscle weakness [1]. Patients with PWS showed reduced total bone mineral density (BMD), bone mineral content (BMC), osteoporosis, and other orthopedic complications [2]. Changes in body composition, including increased fat mass, decreased lean mass, and impaired muscle function, have also been observed in these patients [3,4].

It is known that the musculoskeletal system is mutually coordinated by osteokines and myokines, which are factors secreted by bone cells and skeletal muscle, respectively [5]. Myokines act not only on the skeletal muscle itself and bone metabolism but also mediate the relationships between muscles and other organs and tissues, such as the liver, heart, and adipose tissue, contributing to the pathophysiology of obesity [6]. Many myokines, including irisin, myostatin (MSTN), fibroblast growth factor-2 (FGF-2), insulin growth factor-I (IGF-I), and IGF-binding protein-2 (IGFBP-2), play important roles in the interactions between muscle, bone, and adipose tissue [7].

Irisin is a multifunctional fragment of fibronectin type III domain-containing protein 5 (FNDC5) that promotes the proliferation and differentiation of osteoblasts and inhibits osteoclast differentiation by reducing receptor activator for nuclear factor kappa-B (RANK) expression [8]. In addition, irisin may play a role as an adipocytokine that induces browning of white adipose tissue, but as a myokine, it stimulates myogenic differentiation and inhibits the gene expression of MSTN via the extracellular signal-regulated kinase (ERK) pathway [9,10,11]. MSTN, growth differentiation factor 8 (GDF-8), is a negative regulator of muscle mass that inhibits skeletal muscle proliferation by binding to receptors on the surface of muscle cells [12]. In addition, MSTN leads to a reduction in bone mass due to increased osteoclast formation and intensification of the bone resorption process [13]. Due to the positive association between MSTN and obesity, insulin resistance indices, and metabolic syndrome, this myokine may also be an important factor in the regulation of energy metabolism [14,15].

IGF-I and FGF-2, growth factors produced by skeletal muscle, are positive regulators of bone formation. FGF-2 induces the proliferation and differentiation of osteoblasts and enhances the deposition of greater amounts of mineralized matrix [16]. Muscle-derived IGF-I regulates muscle growth and stimulates bone formation, promoting osteoblast proliferation and improving mature osteoblast function [17]. IGFBP-2 may have an adverse effect on bone metabolism because of the inverse relationships observed between this protein and bone mineral density and appendicular skeletal muscle mass in adults [18].

Previous studies have indicated not only the regulation of bone metabolism by muscle-derived factors, but also the regulation of muscle metabolism by different factors derived from bone, such as osteocalcin (OC) and IGF-I [17,18]. OC secreted by bones affects muscle function and energy metabolism in an endocrine fashion during sedentary activities and exercise [19]. Bone-derived IGF-I regulates the development and regeneration of bone and extra-skeletal organs, including skeletal muscle growth and differentiation [20].

The effects of myokines and osteokines on muscle and bone metabolism are modified by the impact of cytokines derived from adipose tissue. Adipose tissue is an active endocrine organ that secretes various adipokines, such as leptin and adiponectin, which regulate energy metabolism, insulin sensitivity, and inflammation. These adipokines may also influence both bone and muscle metabolism through their endocrine and paracrine actions [6].

Patients with Prader–Willi syndrome appear to have disturbances in muscle, bone, and fat crosstalk. Some authors have analyzed myokine levels in patients with PWS due to muscle weakness, motor problems, and changes in body composition related to the loss of muscle tissue and excess fat tissue [21,22]. However, the results obtained were ambiguous. Circulating irisin was unchanged or lower in individuals with PWS than in controls, but these levels can be influenced by genetic variations, adiposity, and/or vitamin D plasma levels in these patients [22,23,24]. The concentrations of MSTN were studied in obese adults with PWS, and similar levels of this myokine were found compared with healthy individuals [22]. There are limited data on the interplay between muscle, bone, and adipose tissue metabolism in normal-weight children with PWS treated with GH and during low-energy dietary intervention. In our previous study, we observed differences between children with PWS and healthy individuals in the serum concentrations of peptides that regulate appetite [25]. We also showed lower values of total body less head-BMD (TBLH-BMD) and TBLH-BMD Z-scores in these patients together with changes in the profile of biochemical bone turnover markers despite medical and dietary treatment [26]. Because the role of myokines in bone and fat metabolism disorders in patients with PWS is still unclear, the aim of this study was to investigate: (a) the concentrations of selected myokines in patients with PWS during GH treatment and dietary intervention in comparison with healthy normal-weight children following an unrestricted age-appropriate diet; (b) the relationships between the studied myokines, bone turnover markers, and adipokines in patients with PWS; and (c) the relationships between the studied biochemical parameters and body composition in these patients.

## 2. Results

Our results showed similar values of weight, height, and body mass index (BMI) Z-score in both studied groups of children of the same age, but BMI was slightly lower by 7% (*p* = 0.033) in children with PWS than in controls (Table 1).

**Figure 1:**
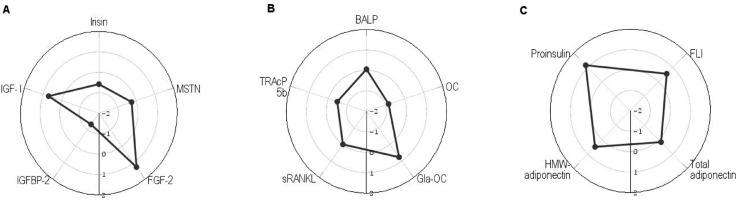
Mean z-scores for children with PWS based on the healthy group: (**A**) myokines, (**B**) bone metabolism markers, and (**C**) adipokines and proinsulin. MSTN—myostatin; FGF-2—fibroblast growth factor-2; IGF-I—insulin-like growth factor-I; IGFBP-2—IGF-binding protein-2; BALP—bone alkaline phosphatase; OC—osteocalcin; Gla-OC—carboxylated-osteocalcin; TRAcP 5b—tartrate-resistant acid phosphatase 5b; sRANKL—soluble receptor activator of nuclear factor kappa-B ligand; FLI—free leptin index; HMW-adiponectin—high molecular weight-adiponectin.

By analyzing myokines concentrations, we found significantly lower values of irisin by approximately 20% (*p* = 0.031) in patients with PWS than in controls, but similar values of MSTN in both the studied groups (Table 1). To visualize the differences between the groups, mean z-scores for the PWS children based on the healthy group are presented on the radar charts (Figure 1). Mean z-scores for children with PWS based on the healthy group were −0.58 for irisin and −0.24 for MSTN (Figure 1A).

Higher levels of IGF-I by approximately 40% (*p* = 0.035) were found in patients with PWS than in healthy children. The median value of FGF-2 was also higher in patients with PWS than in healthy children, but this difference was not statistically significant (*p* = 0.459). The IGFBP-2 concentrations were lower in children with PWS by approximately 30% (*p* < 0.001) compared with the controls. Mean z-scores for children with PWS based on the healthy group were 0.70 for IGF-1, 1.26 for FGF-2, and −1.32 for IGFBP-2 (Figure 1A).

Among the studied bone formation markers, we observed significantly lower concentrations of OC (*p* < 0.001), but significantly higher concentrations of periostin (*p* < 0.001), and a tendency toward higher values of carboxylated-OC (Gla-OC) (*p* = 0.068) in children with PWS than in healthy subjects. Mean z-scores for PWS children based on the healthy group were −0.84 for OC, 1.85 for periostin, and 0.77 for Gla-OC (Figure 1B). In both study groups, similar levels of bone formation marker—bone alkaline phosphatase (BALP)—and bone resorption markers—soluble receptor activator of nuclear factor kappa-B ligand (sRANKL) and tartrate-resistant acid phosphatase 5b (TRAcP 5b)—were found. Mean z-scores for PWS children based on the healthy group were 0.06 for BALP, −0.01 for sRANKL, and −0.47 for TRAcP 5b.

By analyzing the adipokines used, we observed significantly higher values of leptin/soluble leptin receptor (sOB-R) (free leptin index—FLI) (*p* = 0.008) and high-molecular-weight adiponectin (HMW-adiponectin) concentrations (*p* = 0.009) between patients and the control group, but a lack of differences in the total adiponectin concentrations. Mean z-scores for PWS children based on the healthy group were 0.56 for FLI, 0.49 for HMW-adiponectin and −0.17 for total adiponectin. (Figure 1C). Proinsulin levels (*p* = 0.025) were higher in patients with PWS than in healthy subjects, and the mean z-score for PWS children based on the healthy group was 1.15.

**Figure 2:**
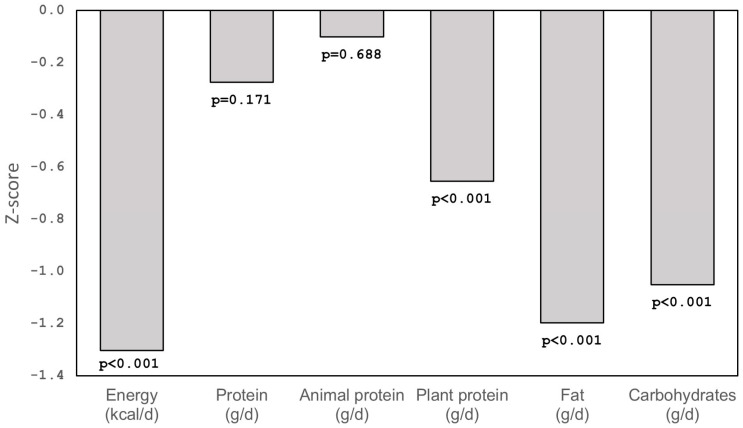
Daily energy and nutrient intake in children with PWS (mean z-scores for children with PWS based on the healthy group).

Daily energy intake was significantly lower in children with PWS than in healthy children, and the mean z-score for the PWS children based on the healthy group was −1.30 (Figure 2).

Total protein and animal protein intakes were similar in both studied groups (mean z-scores for the PWS children based on the healthy group were −0.28 and −0.10, respectively), but plant protein intake was significantly lower in children with PWS than in the control group (mean z-score for the PWS children based on the healthy group was −0.65). Daily intake of fat and carbohydrates was significantly lower in children with PWS than in the control group (mean z-scores for the PWS children based on the healthy group were −1.20 and −1.05, respectively).

Table 2 shows the associations between the studied myokines and anthropometric parameters as well as bone markers in children with PWS. In the partial correlation analysis, we assessed the associations between anthropometric and biochemical parameters in these patients after adjusting for age.

In patients with PWS, positive correlations were observed between irisin concentration and BALP activity (p_partial_ = 0.025), and negative correlations between irisin and Gla-OC (p_partial_ = 0.041) as well as periostin concentrations (p_partial_ = 0.005). FGF-2 concentrations were positively correlated with Gla-OC values (p_partial_ = 0.049). Positive correlations were also found between IGF-I and OC concentrations (p_partial_ = 0.020), as well as between IGF-I and Gla-OC concentrations (p_partial_ = 0.030). IGFBP-2 concentrations were negatively correlated with the height of patients with PWS (p_partial_ = 0.002).

In healthy children, positive correlations were observed in the partial analysis between irisin and OC concentrations (r^S^_partial_ = 0.448, p_partial_ = 0.013), as well as between IGF-I concentration and BALP activity (r_partial_ = 0.526, p_partial_ = 0.007). 

The associations between the studied myokines and adipokines, as well as proinsulin levels in patients with PWS, are shown in Table 3.

In patients with PWS, we observed negative correlations between concentrations of irisin and total adiponectin (p_partial_ = 0.005), and between irisin and HMW-adiponectin concentrations (p_partial_ = 0.014). Positive associations were found between MSTN and HMW-adiponectin (p_partial_ = 0.038), and between MSTN and proinsulin concentrations (p_partial_ = 0.001). IGFBP-2 was positively correlated with HMW-adiponectin levels (p_partial_ = 0.043). In healthy children, positive correlations in the partial analysis were observed between irisin and proinsulin concentrations (r_partial_ = 0.421, p_partial_ = 0.036), and negative correlations between IGFBP-2 and BMI values (r_partial_ = −0.426, p_partial_ = 0.034).

We analyzed body composition in 18 patients with PWS (girls/boys: 10/8) and in 18 healthy children (girls/boys: 11/7) with similar values of BMI (15.2 ± 1.6 vs. 15.6 ± 1.3 kg/m^2^, respectively; *p* = 0.189) and BMI Z-score (−0.57 ± 0.60 vs. −0.40 ± 0.36, respectively; *p* = 0.229). In children with PWS, lean mass was significantly lower by 16% (*p* = 0.047) than in the controls (Table 4).

In addition, we found an approximately 10% higher mean fat mass in patients with PWS than in controls, but this difference was not statistically significant (*p* = 0.092). However, the fat mass/lean mass ratio (*p* < 0.001) was significantly higher in patients than in healthy children. Lower TBLH-BMD Z-scores (*p* = 0.040) were found in the patient group than in the controls.

We assessed the relationships between myokines and body composition in patients with PWS (Table 5).

We found negative correlations between MSTN and lean mass (p_partial_ = 0.015), but positive correlations between IGF-I and TBLH-BMD Z-score (p_partial_ = 0.010) in children with PWS. In healthy children, negative correlations in partial analysis were observed between FGF-2 as well as IGFBP-2 concentrations and lean mass (r = −0.898, p_partial_ < 0.001; r = −1.000, p_partial_ < 0.001, respectively). Negative correlation between IGFBP-2 and BMC values was also found in the controls (r = −0.631, p_partial_ = 0.007).

In addition, we analyzed the relationships between bone metabolism markers, adipokines, and body composition in the patient group. Positive correlations were found between HMW-adiponectin and lean mass (r = 0.586, p_partial_ = 0.013), and between OC and lean mass (r = 0.478, p_partial_ = 0.052) (at the borderline). We observed positive correlations between OC (r = 0.651, p_partial_ = 0.005), Gla-OC (r = 0.576, p_partial_ = 0.016), and periostin (r = 0.786, p_partial_ < 0.001) concentrations and TBLH-BMC values in children with PWS. Positive associations were found between OC and the TBLH-BMD Z-score (r = 0.860, p_partial_ < 0.001), and between FLI and the fat mass/lean mass ratio (r = 0.615, p_partial_ = 0.009) in these subjects.

In healthy individuals, we observed a positive correlation between OC and lean mass (r = 0.651, p_partial =_ 0.005) and a negative correlation between TRAcP 5b values and TBLH-BMC (r = −0.632, p_partial_ = 0.007).

## 3. Discussion

In non-obese children with PWS undergoing growth hormone therapy and dietary intervention, we observed differences in myokine profile and changes in body composition compared with healthy children. We found lower serum concentrations of irisin with lower lean mass and negative correlations between MSTN and lean mass in the studied children with PWS in comparison with healthy individuals. It is known that irisin is an important pro-myogenic factor that stimulates genes related to muscle growth in humans and exerts a beneficial effect on glucose and lipid metabolism, ensuring the maintenance of homeostasis of the musculoskeletal system [23,27]. According to Mai et al. [28] and Hirsch et al. [29], patients with PWS had similar values of irisin compared with healthy individuals. In contrast, Faienza et al. [24] observed lower concentrations of this myokine, suggesting that it may be related to the genetic background and lack of vitamin D supplementation in these patients. The children with PWS in our previous and presented studies received vitamin D supplementation and the serum level of 25-hydroxyvitamin D was significantly higher than in healthy individuals (34.8 ± 9.8 vs. 26.5 ± 8.0 ng/mL) [26]. It cannot be ruled out that genetic factors are responsible for the lower blood concentrations of irisin in patients with PWS. Similar to other authors, we found abnormal body composition, typically lower lean mass, and impaired muscle function in children and adults with PWS [24,28,30]. Positive or negative correlations between irisin and fat-free mass in patients with PWS were observed [23,24], but similar to Mai et al. [28], we did not find such associations.

In contrast to irisin, MSTN inhibits skeletal muscle growth by binding to the activin type IIB myostatin receptor, thereby inhibiting pathways that mediate differentiation in myoblasts and hypertrophy in myotubes [12,31]. Similar to previous studies concerning MSTN in patients with PWS [22,32], we did not find a difference in the levels of this myokine between children with PWS and healthy individuals. However, we observed a negative relationship between MSTN and lean mass in these patients. Due to the central role of MSTN in controlling muscle growth, this myokine has been associated with the loss of muscle mass in sarcopenia, muscular dystrophy, and cachexia and is currently being investigated as a possible therapeutic target in these pathological conditions [33,34]. It seems that the lower levels of irisin and negative association of MSTN with lean mass affect muscle growth processes and consequently be one of the reasons for the lower lean mass observed in the studied children with PWS.

It is known that myokines and osteokines form a network of endocrine-like signals that mediate communication between muscles and bones [35]. Among the osteokines that play an important role in lean muscle metabolism is the bone formation marker—osteocalcin (vitamin K-dependent protein, VKDP) [36]. Osteocalcin affects muscle function and the energy metabolism required for exercise adaptation. This bone marker acts directly on muscle myofibers to increase IL-6 expression, and IL-6 then stimulates a multistep pathway involving gene expression in bone cells, releasing bioactive osteocalcin [37]. In fact, we observed a positive correlation between OC and lean mass in children with PWS, and a much stronger correlation in healthy children. However, we found lower concentrations of total osteocalcin together with abnormal profiles of the osteocalcin forms—Gla- and Glu-OC—in children with PWS than in healthy subjects [26]. In the present study, we also observed negative associations between VKDPs and irisin in children with PWS.

Irisin functions as a myokine but might be considered one of the bone formation markers during childhood [5]. The authors found positive correlations between irisin and osteocalcin, indicating that this myokine forms the bridge between muscle and bone in healthy children. In our study, we also found a correlation between irisin and osteocalcin in healthy individuals, but not in children with PWS. Moreover, Colaianni et al. [5] found that irisin was a greater determinant of bone mineral status than the bone formation marker—BALP. In our patients with PWS, a positive association was observed between irisin and BALP. Irisin regulates bone metabolism by increasing the expression of activating transcription factor 4 (ATF4), runt-related transcription factor 2 (RUNX2), and alkaline phosphatase (ALP), indicating that this myokine may promote osteoblast proliferation and differentiation through the p38/ERK signaling pathways [17,38]. According to Zerlotin et al. [39], a reduction in serum irisin levels may participate in processes related to secondary osteoporosis in humans. We suggest that lower concentrations of irisin and osteocalcin may predispose children with PWS to disturbances in the musculoskeletal system.

FGF-1 and FGF-2 are key factors for maintaining muscle mass and bone density [40]. During GH therapy, we observed higher concentrations of IGF-I and lower IGFBP-2 concentrations in patients with PWS than in the controls, but none of these parameters correlated with lean mass. We found positive correlations between IGF-I and bone formation markers (OC, Gla-OC) and TBLH-BMD Z-scores. In a previous study, we suggested that more intensive carboxylation processes of VKDPs may occur in children with PWS, causing higher concentrations of Gla-OC and periostin in these patients [26]. According to Fang et al. [41], IGF-I deficiency or IGF-I excess may influence bone metabolism and affect bone mass as well as skeletal development. Therefore, the relationships between the GH/IGF-I axis, FGF-2, and VKDPs observed in children with PWS may be related to bone metabolism disorders in these patients.

Adipose tissue contributes to the endocrine control of bone-skeletal muscle crosstalk by releasing adipokines, which may affect both secretory organs [6]. Despite the normal weight and BMI observed in our children with PWS, we found a higher fat mass/lean mass ratio and a tendency toward higher fat mass in these patients than in healthy individuals. In addition, we observed a positive association between FLI and the fat mass/lean mass ratio in non-obese children with PWS. An increased fat mass/lean mass ratio may occur even when normal body weight was achieved in patients with PWS [42]. In our study, we found unchanged total adiponectin concentrations but higher concentrations of HMW-adiponectin in children with PWS. Higher adiponectin concentrations were reported in children [43,44] and adults with PWS [30,45,46] compared with controls. We observed positive associations between MSTN and HMW-adiponectin as well as proinsulin in children with PWS. Altered MSTN and adipokine secretion may contribute to several clinical complications observed in individuals with PWS, including insulin resistance/diabetes, and is implicated in visceral fat accumulation, low muscle mass, and muscle weakness [15,47,48]. Imbalances in myokines, such as MSTN and irisin, together with changes in adipokine levels, may promote a sarcopenic-obesity phenotype [49]. We observed negative correlations between irisin and total adiponectin, and HMW-adiponectin in children with PWS. The reverse relationship between adiponectin and irisin was found in obese children by Karampatsou et al. [50] and Nigro et al. [51]. Irisin is expressed primarily in skeletal muscle and adipose tissue, where it plays a role in insulin sensitivity, increases glucose metabolism, and contributes to energy expenditure and metabolic regulation in browning white adipocytes [52]. The reduction in irisin concentrations with increased adiponectin in children with PWS may suggest a feedback mechanism to decrease energy expenditure in these patients during lifestyle interventions. Monitoring the myokine/adipokine profile could help identify higher risk of a metabolically unhealthy obesity phenotype developing in children with PWS during GH therapy and nutritional management.

Some limitations of the present study should be acknowledged. First, the associations between myokines, bone markers, and adipokines in children with PWS were assessed in a small group of patients due to the rarity of this syndrome in the population. However, all children with PWS during GH therapy and lifestyle intervention were non-obese and similar to the control group in terms of age, sex, and BMI Z-score. Second, body composition analyses using the densitometric method were performed on smaller subgroups of the study population than biochemical analyses. However, both subgroups were representative of the overall groups in terms of age and BMI-related measurements. Third, the cross-sectional nature of the study does not allow for causal inferences or assessment of longitudinal changes in biochemical markers or body composition in response to growth hormone therapy and dietary intervention. Long-term follow-up studies are needed to better understand the progression of musculoskeletal and metabolic changes in PWS over time. Finally, we did not assess the influence of nutritional factors, particularly protein and micronutrient intake, on lean mass and bone metabolism in patients with PWS. Further research should consider these issues in both children and adults with Prader–Willi syndrome during dietary interventions.

## 4. Materials and Methods

### 4.1. Patients

The study involved 52 children aged 2–14 years, including 26 patients with PWS and 26 healthy children, recruited between 2023 and 2025 during dietary counseling at the Institute of Mother and Child in Warsaw. As described in a previous study, the included patients had a genetically confirmed diagnosis of PWS and were undergoing GH treatment for at least one year [25]. All patients were on a low-energy diet with a balanced distribution of carbohydrates, proteins, and lipids, according to the dietary guidelines described previously [25]. Nutritional analysis software Dieta 5^®^ (extended version Dieta 5.0, National Food and Nutrition Institute, Warsaw, Poland) was used to calculate average daily food rations and their nutritional value [53]. The exclusion criteria from the study for both patients and controls were: (a) a body mass index (BMI) Z-score > 1; (b) the use of vitamin and mineral supplements, except vitamin D; (c) the presence of a chronic secondary illness such as diabetes mellitus, liver or kidney diseases, and other chronic diseases with a possible impact on bone metabolism; and (d) not signing the informed consent form.

All patients underwent a general clinical examination and anthropometric measurements. The BMI value of each child was converted to a BMI Z-score according to the Polish reference tables [54]. Of the studied patients, 36 children aged 2–14 years underwent densitometric examinations (18 patients with PWS and 18 healthy children). Body composition was measured using dual-energy X-ray absorptiometry (DXA) using Lunar Prodigy with pediatric software version 9.30.044 (General Electric Healthcare, Madison, WI, USA). Written informed consent was obtained from the parents of all the examined children. The study was performed in accordance with the Helsinki Declaration for Human Research, and the study protocol was approved (protocol code: 17/2022; date of approval: 5 May 2022) by the Ethics Committee of the Institute of Mother and Child in Warsaw, Poland.

### 4.2. Biochemical Methods

Venous blood samples were drawn under fasting conditions between 8:00 and 10:00 a.m., centrifuged at 1000× *g* for 10 min at 4 °C, and stored at −70 °C until required. All studied biochemical parameters were determined using immunoenzymatic methods. The level of MSTN was determined using Quantikine GDF-8/Myostatin Immunoassay ELISA (R&D systems, Minneapolis, MN, USA). The intra- and inter–assay CVs were less than 5.4% and 6.0%, respectively. The mean minimum detectable dose of MSTN was 2.25 pg/mL. ELISA kit from BioVendor (Brno, Czech Republic) was used to measure irisin concentration. The intra- and inter–assay CVs were less than 8.2% and 9.7%, respectively, and the detection limit was 1.0 ng/mL. The concentration of FGF-2 was measured using Human bFGF/FGF2 ELISA Kit from Elabscience Biotechnology (Houston, TX, USA). The intra- and inter–assay CVs were less than 5.4% and 5.7%, respectively, and the sensitivity detection limit was 18.75 pg/mL. Commercially available ELISA kits from Mediagnost (Reutlingen, Germany) were used to determine the concentrations of IGF-I and IGFBP-2. The intra- and inter-assay coefficients of variation were less than 6.7% and 6.6% for IGF-I and below 10% for IGFBP-2, respectively. The analytical sensitivity for IGF-1 was 0.091 ng/mL, and for IGFBP-2 was 0.2 ng/mL.

BALP activity was estimated using the BAP EIA kit from Quidel (Athens, OH, USA), with a within-assay variability of less than 5.8% and a between-assay variability of less than 7.6%. OC concentrations were measured with the N-MID Osteocalcin ELISA kit (IDS, Bolton, UK). The intra-and inter-assay coefficients of variation were less than 2.2% and 5.1% for OC, respectively. The lowest levels of BALP and OC that can be detected by these assays were 0.7 U/L and 0.5 ng/mL, respectively. Gla-OC serum levels were measured using a kit from Takara Bio Inc. (Shiga, Japan), which had intra- and inter-assay CVs of less than 4.8% and 2.4%, respectively. For Gla-OC, the detection limit was 0.25 ng/mL. Periostin levels were determined using the ELISA kit from AdipoGen Life Science (Liestal, Switzerland). Intra- and inter-assay variations were less than 8.6% and 9.9%, respectively, and the detection limit was 15 pg/mL. sRANKL was analyzed using Human sRANKL kits from SunRed Biotechnology (Shanghai, China), with a limit of detection of 1.56 pg/mL. The intra-assay and inter-assay CVs were less than 9% and 11%, respectively. TRAcP 5b was measured using the Bone TRAP ELISA kit (Immunodiagnostic Systems, The Boldons, UK) with the intra-assay and inter-assay CVs less than 9.6% and 9.2%, respectively. The limit of quantitation was less than 0.5 U/L.

ELISA kits from DRG Diagnostics (Marburg, Germany) were used to determine leptin and sOB-R concentrations. The intra- and inter-assay CVs were less than 9.6% and 9.1% for leptin, and 7.2% and 9.8% for sOB-R, respectively. The detection limit was 0.7 ng/mL for leptin. The free leptin index (FLI), calculated as the ratio of leptin to sOB-R concentrations, has been proposed as a marker of free leptin in the circulation [55,56]. Serum levels of total adiponectin and HMW-adiponectin were determined using an ELISA kit (ALPCO Diagnostics, Salem, NH, USA). The intra- and inter-assay variations for total adiponectin were less than 5.4% and 5.0%, respectively. The intra- and inter-assay variations for HMW- adiponectin were less than 5.0% and 5.7%, respectively. The limit of quantitation was 0.019 ng/mL. The concentrations of proinsulin were measured using a kit from TECO Medical (Sissach, Switzerland). The intra- and inter-assay variations were less than 2.2% and 4.0%, respectively. The limit of quantitation was 0.3 pmol/L.

### 4.3. Statistical Analyses

The Kolmogorov–Smirnov test was used to evaluate the distribution for normality. The obtained results are presented as means ± standard deviation (SD) for normally distributed data or medians and interquartile ranges (25th–75th percentiles) for non-normally distributed variables. Differences in anthropometric characteristics, dietary intake, myokines, bone turnover markers, and adipokines between children with PWS and healthy children were assessed using the exact Mann–Whitney test.

Bivariate relationships were assessed using Pearson’s correlation for normally distributed variables and Spearman’s correlation for non-normally distributed variables. To adjust for age, partial correlations (Pearson’s or Spearman’s) were estimated. A *p*-value of <0.05 was considered to be statistically significant. Statistical analysis was performed using IBM SPSS v.29.0 software (SPSS Inc., Chicago, IL, USA) and R ver. 4.1.0 (function *pcor.test*, package *ppcor*).

## 5. Conclusions

In this study of non-obese children with Prader–Willi syndrome undergoing growth hormone therapy and dietary intervention, we observed significant differences in myokine, osteokine, and adipokine profiles, as well as in body composition, compared with healthy peers. Lower circulating levels of irisin and osteocalcin (along with an altered OC form profile), and a negative correlation between MSTN and lean mass, suggest an impaired muscle-bone signaling axis in PWS. These alterations may contribute to deficits in muscle growth and bone metabolism, potentially predisposing children with PWS to future musculoskeletal complications despite GH therapy. Furthermore, disturbances in irisin, MSTN, and adiponectin levels—factors involved in muscle-adipose tissue crosstalk—may indicate an increased risk for the development of metabolic disorders in patients with PWS later in life. Analysis of the myokine/adipokine profile in clinical practice may be helpful in the early detection of metabolic disorders and enable the implementation of treatment aimed at improving health outcomes in patients with Prader–Willi syndrome.

## Figures and Tables

**Table 1:** Anthropometric parameters and biochemical measurements in children with PWS and healthy children.

	Children with PWS n = 26	Healthy Children n = 26	*p*-Values
Age (years)	6.6 ± 3.3	7.6 ± 3.3	0.190
Girls/boys	15/11	15/11	
Height (cm)	116.1 ± 23.6	125.0 ± 19.1	0.131
Weight (kg)	21.5 ± 9.5	25.7 ± 8.7	0.072
BMI (kg/m^2^)	15.0 (14.2–15.9)	16.1 (15.0–16.8)	0.033
BMI Z-score	−0.56 ± 0.68	−0.35 ± 0.38	0.126
Irisin (µg/mL)	3.24 ± 1.46	4.06 ± 1.42	0.031
MSTN (ng/mL)	1.74 (1.42–2.09)	2.07 (1.57–2.48)	0.115
FGF-2 (pg/mL)	44.4 (9.3–125.3)	26.8 (17.3–49.5)	0.459
IGFBP-2 (ng/mL)	254.7 ± 132.4	358.8 ± 78.8	<0.001
IGF-I (ng/mL)	297.7 ± 150.6	217.5 ± 115.3	0.035
BALP (U/L)	125.5 (97.9–148.1)	120.3 (95.1–143.5)	0.519
OC (ng/mL)	56.5 ± 22.6	87.8 ± 37.3	<0.001
Gla-OC (ng/mL)	37.7 ± 16.8	29.0 ± 11.3	0.068
Periostin (ng/mL)	93.9 ± 38.3	60.6 ± 17.9	<0.001
sRANKL (ng/mL)	825 (324–1695)	926 (582–1478)	0.778
TRAcP 5b (U/L)	10.3 ± 2.8	11.6 ± 2.8	0.106
FLI	0.10 (0.05–0.17)	0.03 (0.02–0.09)	0.008
Total adiponectin (µg/mL)	11.6 (9.5–13.8)	9.9 (8.3–12.4)	0.263
HMW-adiponectin (µg/mL)	6.13 (4.52–7.05)	3.97 (3.53–5.34)	0.009
Proinsulin (pmol/L)	1.95 (1.56–2.90)	1.35 (0.91–2.24)	0.025

Data are presented as mean ± SD or median values (25th–75th percentiles). BMI—body mass index; MSTN—myostatin; FGF-2—fibroblast growth factor-2; IGF-I—insulin-like growth factor-I; IGFBP-2—IGF-binding protein-2; BALP—bone alkaline phosphatase; OC—osteocalcin; Gla-OC—carboxylated osteocalcin; TRAcP 5b—tartrate-resistant acid phosphatase 5b; sRANKL—soluble receptor activator of nuclear factor kappa-B ligand; FLI—free leptin index; HMW-adiponectin—high molecular weight-adiponectin.

**Table 2:** Relationships between myokines, anthropometric parameters, and bone turnover markers in the studied children with PWS.

	Correlation Coefficient	Irisin	MSTN	FGF-2	IGF-I	IGFBP-2
Height	bivariate (p) partial * (p)	−0.043 (0.835) 0.064 (0.759)	0.282 (0.162) −0.230 (0.268)	0.485 (0.012) 0.289 (0.161)	0.810 (<0.001) 0.169 (0.418)	−0.731 (<0.001) −0.590 (0.002)
Weight	bivariate (p) partial * (p)	−0.011 (0.956) 0.129 (0.539)	0.297 (0.141) −0.088 (0.675)	0.400 (0.043) −0.010 (0.962)	0.770 (<0.001) 0.060 (0.775)	−0.645 (<0.001) −0.195 (0.350)
BMI	bivariate (p) partial * (p)	0.160 (0.436) 0.182 (0.384)	0.079 ^S^ (0.701) 0.031 ^S^ (0.883)	−0.192 ^S^ (0.348) −0.299 ^S^ (0.147)	0.127 ^S^ (0.535) 0.013 ^S^ (0.952)	−0.083 ^S^ (0.685) −0.195 ^S^ (0.350)
BMI Z-score	bivariate (p) partial * (p)	0.136 (0.507) 0.137 (0.515)	0.007 (0.973) 0.006 (0.976)	−0.216 (0.289) −0.241 (0.245)	−0.124 (0.547) −0.217 (0.298)	0.077 (0.707) 0.103 (0.624)
BALP	bivariate (p) partial * (p)	0.427 (0.029) 0.447 (0.025)	0.006 (0.978) −0.064 (0.763)	−0.020 (0.923) −0.112 (0.595)	0.270 (0.183) 0.211 (0.312)	0.001 (0.994) 0.155 (0.461)
OC	bivariate (p) partial * (p)	−0.157 (0.444) −0.145 (0.490)	0.049 (0.812) −0.075 (0.721)	0.041 (0.841) −0.118 (0.573)	0.521 (0.006) 0.461 (0.020)	−0.075 (0.715) 0.183 (0.382)
Gla-OC	bivariate (p) partial * (p)	−0.414 (0.035) −0.411 (0.041)	0.088 (0.669) 0.013 (0.951)	0.444 (0.023) 0.398 (0.049)	0.424 (0.031) 0.436 (0.030)	−0.092 (0.657) 0.061 (0.771)
Periostin	bivariate (p) partial * (p)	−0.529 (0.005) −0.541 (0.005)	0.007 (0.971) 0.052 (0.805)	0.239 (0.239) 0.324 (0.114)	−0.083 (0.685) 0.020 (0.923)	0.352 (0.078) 0.362 (0.076)
sRANKL	bivariate (p) partial * (p)	0.308 (0.126) −0.349 (0.088)	−0.020 ^S^ (0.925) 0.196 ^S^ (0.349)	−0.380 (0.324) −0.150 (0.473)	−0.521 (0.006) −0.007 (0.973)	0.516 (0.007) 0.189 (0.366)
TRAcP 5b	bivariate (p) partial * (p)	0.038 (0.852) 0.057 (0.787)	0.410 (0.038) 0.351 (0.085)	0.201 (0.324) 0.101 (0.632)	0.277 (0.170) 0.113 (0.591)	−0.092 (0.656) 0.101 (0.631)

(*) age-adjusted, (^S^) Spearman correlation coefficient, otherwise Pearson correlation coefficient. BMI—body mass index; MSTN—myostatin; FGF-2—fibroblast growth factor-2; IGF-I—insulin-like growth factor-I; IGFBP-2—IGF-binding protein-2; BALP—bone alkaline phosphatase; OC—osteocalcin; Gla-OC—carboxylated osteocalcin; TRAcP 5b—tartrate-resistant acid phosphatase 5b; sRANKL—soluble receptor activator of nuclear factor kappa-B ligand.

**Table 3:** Relationships between myokines and adipokines, and proinsulin levels in children with PWS.

	Correlation Coefficient	Irisin	MSTN	FGF-2	IGF-I	IGFBP-2
FLI	bivariate (p) partial * (p)	−0.062 (0.762) −0.033 (0.877)	0.403 (0.041) 0.261 (0.207)	0.001 (0.998) −0.344 (0.093)	0.418 ^S^ (0.034) −0.205 ^S^ (0.327)	−0.521 ^S^ (0.006) −0.124 ^S^ (0.555)
Total adiponectin	bivariate (p) partial * (p)	−0.540 ^S^ (0.004) −0.539 ^S^ (0.005)	0.309 (0.125) 0.295 (0.152)	0.116 (0.572) 0.083 (0.692)	0.140 (0.496) 0.107 (0.610)	0.170 (0.408) 0.299 (0.147)
HMW- adiponectin	bivariate (p) partial * (p)	−0.482 ^S^ (0.013) −0.484 ^S^ (0.014)	0.392 ^S^ (0.048) 0.417 ^S^ (0.038)	0.258 ^S^ (0.203) 0.296 ^S^ (0.150)	0.140 ^S^ (0.496) 0.223 ^S^ (0.285)	0.352 (0.078) 0.407 (0.043)
Proinsulin	bivariate (p) partial * (p)	0.169 ^S^ (0.409) 0.196 ^S^ (0.347)	0.677 ^S^ (<0.001) 0.638 ^S^ (0.001)	0.401 ^S^ (0.042) 0.292 ^S^ (0.156)	0.504 (0.009) 0.350 (0.086)	−0.225 (0.269) 0.030 (0.888)

(*) Age-adjusted, (^S^) Spearman correlation coefficient, otherwise Pearson correlation coefficient. MSTN—myostatin; FGF-2—fibroblast growth factor-2; IGF-I—insulin-like growth factor-I; IGFBP-2—IGF-binding protein-2; FLI—free leptin index; HMW-adiponectin—high molecular weight-adiponectin.

**Table 4:** Densitometric parameters in patients with PWS and healthy children.

	Children with PWS n = 18	Healthy Children n = 18	*p*-Values
Fat mass (kg)	4.93 (4.17–6.61)	4.39 (2.6–4.98)	0.092
Lean mass (kg)	17.7 ± 5.6	21.0 ± 4.7	0.047
Fat mass/lean mass	0.31 (0.25–0.41)	0.18 (0.15–0.22)	<0.001
TBLH-BMC (kg)	0.51 ± 0.15	0.49 ± 0.13	0.932
TBLH-BMD Z-score	−0.71 ± 0.70	−0.24 ± 0.52	0.040

Data are presented as mean ± SD. TBLH—total body less head; BMC—bone mineral content; BMD—bone mineral density.

**Table 5:** Relationships between myokines and densitometric parameters in children with PWS.

	Correlation Coefficient	Irisin	MSTN	FGF-2	IGF-I	IGFBP-2
Fat mass	bivariate (p) partial * (p)	−0.100 (0.693) −0.079 (0.764)	0.029 (0.910) −0.241 (0.351)	0.215 ^S^ (0.392) −0.230 ^S^ (0.374)	0.560 ^S^ (0.016) 0.113 ^S^ (0.666)	−0.485 (0.042) −0.170 (0.513)
Lean mass	bivariate (p) partial * (p)	−0.206 (0.413) −0.373 (0.141)	0.105 (0.678) −0.576 (0.015)	0.454 (0.058) 0.161 (0.536)	0.762 (<0.001) 0.076 (0.771)	−0.559 (0.016) 0.072 (0.784)
Fat/lean mass	bivariate (p) partial * (p)	−0.003 (0.990) 0.005 (0.985)	−0.004 (0.989) −0.054 (0.837)	−0.160 (0.527) −0.243 (0.348)	0.021 (0.933) −0.149 (0.568)	−0.213 (0.396) −0.168 (0.519)
TBLH-BMC	bivariate (p) partial * (p)	−0.300 (0.226) −0.456 (0.066)	0.071 ^S^ (0.779) −0.212 ^S^ (0.413)	0.397 ^S^ (0.102) 0.032 ^S^ (0.902)	0.741 (<0.001) 0.189 (0.467)	−0.627 (0.005) −0.227 (0.381)
TBLH-BMD Z-score	bivariate (p) partial * (p)	−0.130 (0.607) −0.121 (0.645)	0.202 (0.421) 0.147 (0.573)	0.230 (0.358) 0.166 (0.523)	0.503 (0.033) 0.607 (0.010)	−0.011 (0.964) 0.145 (0.579)

(*) Age-adjusted, (^S^) Spearman correlation coefficient, otherwise Pearson correlation coefficient. MSTN—myostatin; FGF-2—fibroblast growth factor-2; IGF-I—insulin-like growth factor-I; IGFBP-2—IGF-binding protein-2; TBLH—total body less head; BMC—bone mineral content; BMD—bone mineral density.

## Data Availability

The data presented in this study are available upon reasonable request to the corresponding author.

## Abbreviations

The following abbreviations are used in this manuscript:

BALP	Bone alkaline phosphatase
BMC	Bone mineral content
BMD	Bone mineral density
BMI	Body mass index
FGF-2	Fibroblast growth factor-2
FLI	Free leptin index
GH	Growth hormone
Gla-OC	Carboxylated osteocalcin
HMW-adiponectin	High molecular weight-adiponectin
IGF-I	Insulin-like growth factor-I
IGFBP-2	IGF-binding protein-2
MSTN	Myostatin
OC	Osteocalcin
PWS	Prader–Willi syndrome
RANK	Receptor activator for nuclear factor kappa-B
sRANKL	Soluble receptor activator of nuclear factor kappa-B ligand
sOB-R	Soluble leptin receptor
TBLH-BMD	Total body less head-BMD
TRAcP 5b	Tartrate-resistant acid phosphatase 5b
VKDP	Vitamin K-dependent protein
